# G-Protein Coupled Receptor Protein Synthesis on a Lipid Bilayer Using a Reconstituted Cell-Free Protein Synthesis System

**DOI:** 10.3390/life8040054

**Published:** 2018-11-02

**Authors:** Belay Gessesse, Takashi Nagaike, Koji Nagata, Yoshihiro Shimizu, Takuya Ueda

**Affiliations:** 1 Department of Computational Biology and Medical Sciences, Graduate School of Frontier Sciences, The University of Tokyo, Bldg. FSB-401, 5-1-5 Kashiwanoha, Kashiwa, Chiba 277-8562, Japan; belaygessesse@gmail.com (B.G.); nagaike@edu.k.u-tokyo.ac.jp (T.N.); 2 Laboratory for Cell-Free Protein Synthesis, RIKEN Center for Biosystems Dynamics Research (BDR), 6-2-3, Furuedai, Suita, Osaka 565-0874, Japan; yshimizu@riken.jp; 3 Department of Applied Biological Chemistry, Graduate School of Agricultural and Life Sciences, The University of Tokyo, 1-1-1 Yayoi, Bunkyo-ku, Tokyo 113-8657, Japan; aknagata@mail.ecc.u-tokyo.ac.jp

**Keywords:** cell-free protein synthesis, artificial cell, lipid bilayer, membrane protein, G-protein coupled receptor, lipid nanodisc

## Abstract

Membrane proteins are important drug targets which play a pivotal role in various cellular activities. However, unlike cytosolic proteins, most of them are difficult-to-express proteins. In this study, to synthesize and produce sufficient quantities of membrane proteins for functional and structural analysis, we used a bottom-up approach in a reconstituted cell-free synthesis system, the PURE system, supplemented with artificial lipid mimetics or micelles. Membrane proteins were synthesized by the cell-free system and integrated into lipid bilayers co-translationally. Membrane proteins such as the G-protein coupled receptors were expressed in the PURE system and a productivity ranging from 0.04 to 0.1 mg per mL of reaction was achieved with a correct secondary structure as predicted by circular dichroism spectrum. In addition, a ligand binding constant of 27.8 nM in lipid nanodisc and 39.4 nM in micelle was obtained by surface plasmon resonance and the membrane protein localization was confirmed by confocal microscopy in giant unilamellar vesicles. We found that our method is a promising approach to study the different classes of membrane proteins in their native-like artificial lipid bilayer environment for functional and structural studies.

## 1. Introduction

The advent of artificial cells has enabled us to study active membrane proteins that play a substantial role in various cellular activities and physiological functions under a defined condition [1,2]. Membrane proteins are expressed and localized to the biological membrane to modulate the functions of the membrane by regulating the bidirectional flux of molecules and cell-to-cell communications. It is estimated that about 30–50% of all pharmaceutical drugs target membrane proteins such as G-protein coupled receptors (GPCRs), ion channels, membrane transporters and enzymes [3,4,5,6].

The synthesis of active membrane proteins using artificial lipid bilayers has a significant advantage in examining the complex biochemical reactions and to screen for drug candidates. Artificial cells have been manipulated to study the functions of membrane proteins such as α-hemolysin [7,8], a potassium channel, KcsA [9] and olfactory receptor complexes [10]. The use of artificial cells can also be extended to other classes of membrane proteins such as GPCRs which can respond to extracellular signals in the environment. Therefore, the synthesis and integration of active GPCRs into artificial lipid bilayer mimetics such as nanodiscs and giant unilamellar vesicles (GUVs) have a substantial role to screen ligands and to develop biosensors for medical applications [11].

Currently, cell-free protein synthesis systems have become an emerging potential bottom-up approach to study membrane proteins for functional and structural analysis. The Protein Synthesis Using Recombinant Elements system (PURE system) is an *E. coli* based cell-free system which contains purified components of transcriptional and translational factors at a defined concentration unlike the total *E. coli* extract based cell-free system [12]. This enables us to control and modify the system more easily as per our objectives. Moreover, the PURE system is devoid of nucleases and proteases and offers improved controllability. To date, various functional membrane proteins were synthesized by the cell-free systems supplemented with detergents, chaperones, micelles, liposomes and nanodiscs. These include C-C Chemokine receptor type 5 (CCR5) [13], claudin-4 [14], secYEG [15] and human endothelin B receptor (ETB) [16].

Incorporation of lipid nanodiscs (NDs) into the cell-free system during membrane protein expression has a greater advantage over vesicle-based mimetics such as GUVs. Nanodiscs are generally soluble, stable, monodisperse and more importantly, both the N- and C-terminus are accessible for ligand binding assays [17,18]. Recently, the nanodisc technology has been used for the reconstitution of various membrane proteins such as a G-protein coupled receptor, CCR5 [19], an ion channel TRPV1 [20] and a transporter, MsbA [21]. In addition to the direct reconstitution of purified membrane proteins into nanodiscs, co-translational integration of membrane proteins by a cell-free system was nonetheless used for adrenergic receptor 1 (β1AR) [22] and endothelial receptor (ETB) [16,23]. From a structural point of view, nanodiscs in combination with cell-free systems will offer a greater advantage in determining the structure of membrane proteins in their native-like lipid bilayer by cryo-electron microscopy (cryo-EM) [21,24].

**Figure 1:**
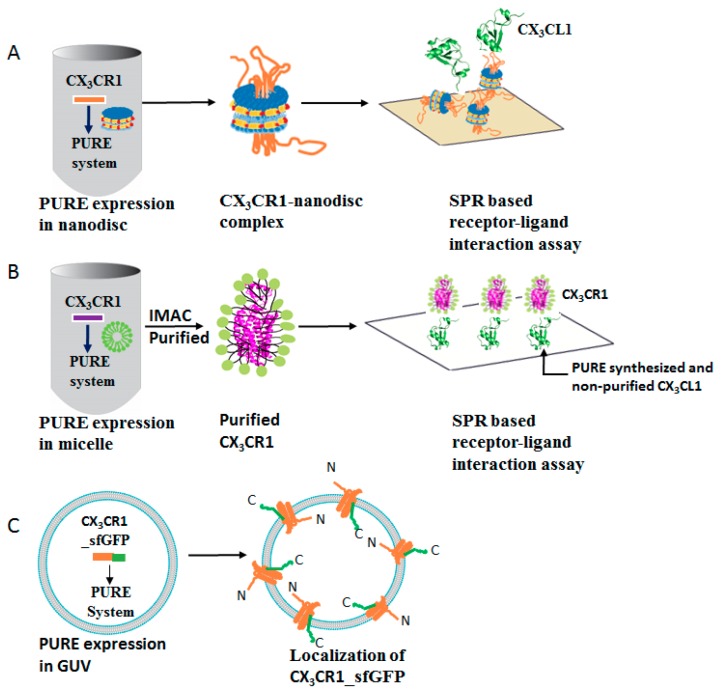
Schematic representation of the cell-free expression of GPCRs. CX_3_CR1 was synthesized by the PURE system supplemented with lipid nanodiscs (**A**), micelle (**B**), or CX_3_CR1-sfGFP synthesized inside GUV (**C**). The GPCR-nanodisc complex was immobilized on a sensor chip without prior purification for Surface Plasmon Resonance (SPR) based ligand binding assay. Similarly, PURE expressed CX_3_CR1 in micelle was purified and allowed to interact with pre-immobilized PURE expressed and non-purified CX_3_CL1 to determine the binding affinity by SPR. The localization of CX_3_CR1-sfGFP was probed by confocal microscopy.

In this study, we used a PURE system based co-translational integration of GPCRs into artificial lipid bilayers and micelles for functional and structural analysis. To gain an insight into the functions of GPCRs, we chose the chemokine GPCRs, CX_3_CR1 and CCR5 as model proteins. We systematically optimized the expression and analyzed the secondary structure, membrane localization, activity and homogeneity of the synthesized chemokine GPCRs by using nanodiscs, GUVs and micelles (Figure 1A–C).

## 2. Materials and Methods

### 2.1. Materials

Synthetic lipids such as 1-palmitoyl-2-oleoyl-glycero-3-phosphocholine (POPC), 1-palmitoyl-2-oleoyl-sn-glycero-3-phospho-l-serine (sodium salt) (POPS), 1,2-distearoyl-sn-glycero-3-phosphoethanolamine-*N*-[methoxy(polyethylene glycol)-2000] (ammonium salt) (PEG2000-DOPE) were purchased from Avanti Polar Lipids, Inc. (Alabaster, AL, USA); Cholesterol from Nacalai tesque, Inc. (Kyoto, Japan); SM2 bio-beads from Bio-rad (Hercules, CA, USA); Penta-His antibody from Qiagen (Hilden, Germany); StrepMAB-Immo from IBA Lifesciences (Goettingen, Germany); CM5 and amine coupling reagents from GE Healthcare (Chicago, IL, USA).

### 2.2. Preparation of DNA Constructs

The cDNAs of human GPCRs, CX_3_CR1 and CCR5, were a kind gift from Dr. Yutaka Suzuki (University of Tokyo). The sequence of CX_3_CR1 was optimized for *E. coli* expression by Genscript whereas the sequence of human CCR5 was used without optimization. Plasmids and linear PCR products of both CX_3_CR1 and CCR5 were constructed and used as a DNA template for protein expression. The PCR fragment of both CX_3_CR1 and CCR5 were generated by two-step overlap PCR using a forward primer containing a T7 promoter and Shine Dalgarno sequence and a reverse primer with or without a His-tag. To enhance the stability of CCR5, a rubredoxin was inserted between amino acid residues Arg223 and Glu227 [25] to produce a stabilized CCR5 variant, CCR5-Rb, in which the template DNA preparation was the same as CX_3_CR1 and CCR5. The expression plasmids of CX_3_CR1, CCR5 and CCR5-Rb were constructed by infusion cloning of the DNA containing 6xhis tag and Tev recognition site preceded with a linker (DYDIPTT) at the N-terminus to pET28a vector digested with NcoI and XhoI restriction enzymes. For localization experiment, CX_3_CR1-sfGFP fusion construct was made by fusing sfGFP to the C-terminus of CX_3_CR1 DNA by overlap PCR and cloned to pET28a vector digested with EcoRI and XhoI restriction enzymes through infusion cloning. According to a previously reported method [26], the ligand fractalkine (CX_3_CL1) containing strep-tag II at the N-terminus was amplified from pUREstrept2 plasmid for immobilization and binding assay. 

### 2.3. Membrane Scaffold Protein (MSP) Expression

The expression and purification of MSP were carried out according to the established protocols with slight modifications [27]. Briefly, the expression host BL21 (DE3) containing either MSP1D1 (addgene#20061) or MSP1E3D1 (addgene#20066) plasmids were expressed in 3 L LB culture medium containing 50 µg/mL kanamycin at 37 °C with shaking and at OD_600_ 0.8–1, the culture was induced with 1 mM IPTG. The culture was further incubated for 1 h at 37 °C and for 3 h at 28 °C to prevent aggregation. The cell pellet was then collected at 8000× *g* for 15 min at 4 °C, washed with MSP buffer containing EDTA, flash frozen in liquid N_2_ and stored at −80 °C. For purification, the MSP was thawed at room temperature for 30 min and suspended with lysis buffer (40 mM Tris/HCl, pH 8.0, 300 mM NaCl) containing 1% Triton X-100, protease inhibitor cocktail and DNase (0.5 mg for 5 gm cell pellet). The supernatant was then centrifuged at 30,000× *g* for 30 min at 4 °C to remove the cell debris and purified by IMAC affinity chromatography using Ni-NTA column pre-equilibrated with lysis buffer containing 1% Triton X-100. Subsequently, the column was washed with buffers A_msp_ (Lysis Buffer containing 1% Triton X-100), B_msp_ (Lysis Buffer containing 50 mM Na Cholate, 20 mM Imidazole), C_msp_ (Lysis Buffer containing 50 mM Imidazole). The MSP protein was later eluted with buffer D_msp_ (Lysis Buffer containing 400 mM Imidazole), analyzed by 15% SDS-PAGE and buffer exchanged with 20 mM Tris/HCl, pH 8.0, 100 mM NaCl and 0.5 mM EDTA in a PD-10 column. Finally, the fraction containing pure protein was collected, concentrated, aliquoted and flash frozen in liquid nitrogen at −80 °C until further use. The protein concentration was determined by absorbance at 280 nm with an extinction coefficient of ε_280_ = 21,000 M^–1^ cm^–1^ and ε_280_ = 29,400 M^–1^ cm^–1^ for MSP1D1 and MSP1E3D1 respectively.

### 2.4. Nanodisc (ND) Preparation

Nanodiscs were prepared according to previous reports with slight modification [28,29,30]. In brief, synthetic lipids POPC, POPS and Cholesterol were dissolved in chloroform and mixed at a ratio of 72%, 20% and 8% respectively. The mixture was then flushed with N_2_ gas and dried overnight in a vacuum. The dried lipid film was solubilized by a nanodisc buffer (20 mM Tris/HCl, pH 7.4 and 100 mM NaCl) containing 100 mM Na cholate. To achieve complete solubilization, the mixture was vortexed followed by heating at 60 °C in a water bath until the lipid film completely dissolved. Finally, detergent solubilized lipid was mixed either with MSP1D1 or MSP1E3D1 at the respective molar ratio of 1:60 and 1:85 respectively and incubated at 4 °C. After 1 h of incubation, an adsorbent, SM2 bio-beads from Bio-rad (Hercules, CA, USA), was added to remove the detergent and to facilitate nanodisc formation. The reaction mixture was further incubated from 6 h to overnight and the Biobeads were removed by centrifugation at 130,000× *g* for 2 min using a 0.2 µm NANOSEP device from PALL Life Sciences (Port Washington, NY, USA). To remove the aggregates, the nanodisc was further centrifuged at 18,000× *g* for 5 min before loading to Superdex-200 10/300 from GE Healthcare (Chicago, IL, USA) for further purification.

### 2.5. PURE Synthesis of Membrane Proteins

A PURE system (PUREfrex 2) prepared according to published protocols [31] and purchased from Genefrontiers (Kashiwa, Japan) were used for membrane protein synthesis. To facilitate correct disulfide bond formation, we used a modified PURE system in which the DTT was replaced by glutathione reduced (GSH). A GPCR was synthesized from 5 nM DNA template by the PURE system in the presence or absence of nanodiscs in a 20 µL reaction volume and incubated at 37 °C for 6 h. After the reaction, the synthesized membrane protein was aliquoted into 5 µL as a total fraction. The remaining 15 µL was centrifuged at 20,400× *g* for 10 min at 4 °C and the supernatant was collected. A sample loading dye was added at a 1:1 (*v*/*v*) ratio to the total and supernatant fraction and run on 15% SDS-PAGE. For radiolabeling based quantification, ^35^S-methionine incorporation by the PURE system into the membrane protein was measured by BAS-5000 from Fujifilm (Tokyo, Japan) and the band intensity was quantified by Multi Gauge software (Tokyo, Japan).

### 2.6. Purification of Membrane Protein-Nanodisc Complex

After PURE synthesis, the reaction mixture was centrifuged at 18,000× *g* for 3 min at 4 °C prior to purification. The supernatant was mixed with Ni-NTA equilibrated with buffer A_cplx_ (20 mM Tris/HCl, pH 8.0, 150 mM NaCl) and incubated at 4 °C for 1 h with shaking. The column was washed with wash buffer B_cplx_ (buffer A containing 30 mM Imidazole) and the nanodisc-membrane protein complex was eluted with buffer C_cplx_ (buffer A containing 300 mM Imidazole. The elution fraction was run on SDS-PAGE to monitor the purity of the purification and buffer exchanged by dialysis with the elution buffer without imidazole, concentrated and quantified by A_280_ absorbance. For transmission electron microscopy, the elution fraction after IMAC purification was diluted 1:1 (*v*/*v*) in nanodisc buffer and further purified by size exclusion chromatography in a Superdex-200 10/300 column (GE Healthcare) using a nanodisc buffer.

### 2.7. Circular Dichroism (CD) Spectroscopy

Far-UV CD measurements were performed on J720 spectropolarimeter from Jasco (Tokyo, Japan) using a Teflon sealed 1 mm path length quartz glass cuvette from Hellma Analytics (Mullheim, Germany). A wavelength increment of 1 nm, a response time of 4 s, a scan speed of 20 nm/min parameters and a concentration of 0.15 mg/mL of CX_3_CR1 and 0.11 mg/mL of CCR5-Rb were used during measurement. All the resulting spectra were buffer corrected.

### 2.8. Preparation of Membrane Proteins for CD Spectroscopy by Micelle Method

Mixed Micelles were prepared as described in Shinoda et al. [14] from 25 mg/mL brain polar lipid and 75 mg/mL of digitonin detergent and tip sonicated until the mixture becomes transparent. The micelle at 0.15 volume of reaction was added to a PURE based cell-free system and the reaction mixture was incubated for 4 h at 37 °C. After protein synthesis, the mixture was centrifuged at 100,000× *g* for 30 min at 4 °C and the supernatant was applied to Ni-NTA pre-equilibrated with buffer A (20 mM Tris/HCl, pH 8, 150 mM NaCl, 0.05% DDM and 0.002% CHS) and incubated at 4 °C for 1 h with gentle shaking. IMAC purification was carried out by washing the column in buffer B (buffer A containing 20 mM Imidazole) and the recombinant protein was eluted with buffer C (buffer A supplemented with 300 mM Imidazole). The eluate was then concentrated and buffer exchanged with buffer A by repeated dilute and concentrate method in a 10 kDa MWCO from Merck Millipore (Tullagreen, Ireland). The concentration of the protein was finally quantified by A_280_ absorbance in nanodrop 1000 from Thermo Fisher Scientific (Waltham, MA, USA) and used for CD measurement.

### 2.9. Electron Microscopy of CX_3_CR1-Nanodisc Complexes

IMAC purified CX_3_CR1-nanodisc complexes were further purified by size exclusion chromatography and concentrated to 0.25 mg/mL using a 30 kDa molecular weight cutoff (Merck Millipore). About 2 µL of the CX_3_CR1-nanodisc complex at 100 nM was deposited on a glow-discharged copper grid, incubated for 1 min and blotted away using a filter paper. Immediately after blotting, the grid was stained with 5 µL of 2% (*w*/*v*) uranyl acetate and blotted away after 30 s incubation at room temperature. The spotted sample was then dried at room temperature or using a lamp for quick drying and electron micrographs were recorded on H-7000 electron microscopy from Hitachi (Tokyo, Japan) operated at an acceleration voltage of 100 kV and at 30,000× magnification.

### 2.10. Giant Unilamellar Vesicle (GUV) Preparation

Giant unilamellar vesicle preparation was carried out according to established protocols with slight modification [8]. Briefly, POPC lipid or a mixture of POPC and PEG2000PE (9.75: 0.25 molar ratio) was mixed, at the concentration of 10 mM, with 500 µL liquid paraffin. The mixture was vortexed vigorously, flushed with N_2_ gas and heated at 80 °C for 20 min. The heated lipid-paraffin mixture was vortexed until it gets cooled. The lipid-paraffin mix was flushed with N_2_ gas again and subjected to water bath sonication for at least 30 min at 55 °C. The lipid-paraffin mixture was allowed to cool at room temperature and 300 µL of the mixture was transferred to a small glass tube. The inner solution mixture, composed of the PURE system, 200 mM sucrose and the template DNA in a 30 µL of reaction volume, was added at the bottom of the cooled lipid-paraffin mix. The emulsion was later formed by very brief and gentle pipette in-and-out. The prepared emulsion was then overlaid at the top of a 200 µL ice-chilled outer solution consisting of the PURE buffer without tRNA and 200 mM glucose in a 1:1 (*v*/*v*) ratio. The mixture was further kept on ice for 10 min and centrifuged for 30 min at 10,000× *g* at 4 °C. The precipitated GUV was collected by purging the Eppendorf tube at the bottom with 21 G × 1 ½-inch needle. RNase was added to the collected supernatant at the concentration of 20 ng µL^−1^ and the GUV suspension was incubated at 37 °C for 6–8 h. Finally, the localization of the sfGFP fused CX_3_CR1 was analyzed by confocal imaging. For sfGFP expression, the GUV was prepared from POPC and PEG2000-DOPE lipids.

### 2.11. Determination of Binding Constants by the Surface Plasmon Resonance (SPR)

SPR based ligand binding was carried out using Biacore T200 (GE Healthcare). Penta-His antibody (Qiagen) and strepMAB-Immo antibody (IBA Lifesciences) were immobilized on CM5 sensor chip using the standard amine coupling chemistry to capture His-tagged CX_3_CR1-nanodisc complex and cell-free synthesized CX_3_CL1 containing Strep-tag II respectively. In brief, flow cell one, which was used as reference and flow cell two of the sensor chip were activated for 7 min with a 1:1 mixture of 0.4 M EDC (1-ethyl-3-(3-dimethylaminopropyl)-carbodiimide) in water and 0.1 M NHS (N-hydroxysuccinimide) in water at a flow rate of 10 µL/min. About 9000 RU of Penta-His and 1000 RU of StrepMAB-Immo antibody at 50 µg/mL in 10 mM sodium acetate, pH 5.0 were immobilized for 7 min at a flow rate of 10 µL/min in a running buffer (HBS-P) containing 10 mM HEPES, pH 7.4, 150 mM NaCl, 0.05% surfactant P20 for the nanodisc and micelle system respectively. To deactivate excessive reactive groups, the surfaces of both flow cells were blocked with a 1 M ethanolamine, pH 8.5. After 6 h of incubation at 37 °C, cell-free synthesized His-tagged CX_3_CR1in nanodisc (~300 kDa) and CX_3_CL1-tagged with Strep-tag II (8.5 kDa) were directly immobilized to flow cell two up to ~1500 RU and ~1000 RU respectively. For the nanodisc system, purified CX_3_CL1 diluted in the running buffer was injected on both flow cells at a concentration of 5 nM, 10 nM, 20 nM, 40 nM and 80 nM and at a flow rate of 30 µL/min in the order of increasing concentration at 25 °C. The association and dissociation rate was set at 120 and 300 s, respectively. For the micelle system, PURE expressed CX_3_CR1in micelle was diluted in the running buffer and injected to both flow cells with the same condition as the nanodisc system. A 10 mM glycine/HCl, pH 1.5 was used for surface regeneration. The K_D_ value was calculated with the Biacore T200 evaluation software version 3.0 using a 1:1 interaction model.

### 2.12. Confocal Microscopy

Confocal fluorescence microscopy was performed with an objective lens of 63×. After PURE synthesis, 5 µL of the sample was aliquoted and mixed with Nile red staining dye (100 µM) and both green and blue channels were selected for taking images. Upon taking the images, the sensitivity of the laser was adjusted optimum by Zeiss software.

## 3. Results

### 3.1. Membrane Protein Synthesis in Lipid Nanodisc

**Figure 2:**
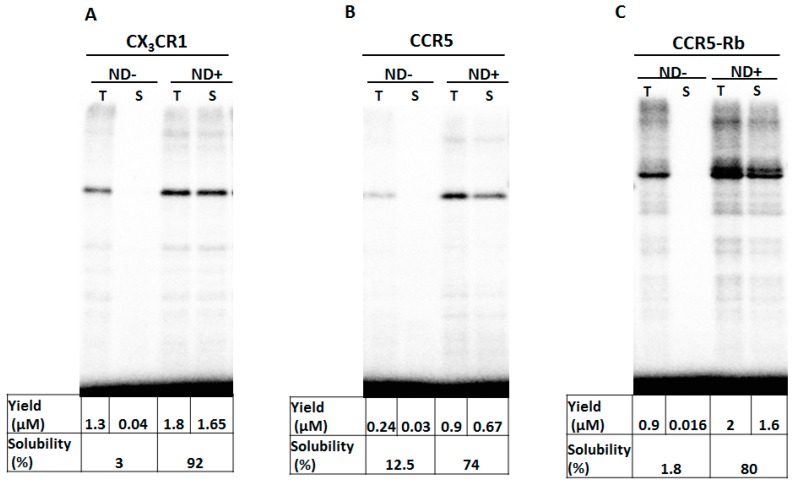
Productivity and solubility of cell-free expressed GPCRs. The GPCRs were expressed in the PURE system containing [^35^S]-Methionine in the presence or absence of nanodisc (ND+ or ND-, respectively). The productivity and solubility were subsequently quantified for CX_3_CR1 (**A**), CCR5 (**B**) and CCR5-Rb (**C**). The soluble fraction was quantified by dividing the supernatant (S) by the total (T) amount of synthesized protein.

To understand the functional and structural roles of a repertoire of GPCRs, we systematically examined their expression and productivity by the PURE system containing lipid nanodiscs. Due to the possibility of manipulating the PURE system reaction condition, we added nanodiscs to facilitate solubilization and stability of membrane protein interests. In this particular experiment, we supplemented the PURE system with lipid bilayers such as nanodiscs and evaluated the expression of the G-protein coupled receptors CX_3_CR1 and CCR5. In addition, since previous reports have shown that the insertion of rubredoxin, an iron-sulfur redox protein, into the third intracellular loop of CCR5 enhanced the thermostability [25,32], we tested the expression of a fusion protein where rubredoxin was inserted to CCR5 (CCR5-Rb). Nanodiscs have been used to improve the stability and solubility of membrane proteins and widely used to study membrane proteins through reconstitution [30] and co-translational integration [33]. Consequently, the insertion and synthesis of membrane proteins are affected by the nanodiscs and incorporating the right size and concentration of nanodisc is essential. Thus, we investigated the effect of nanodiscs on the solubility of membrane proteins, which indicated the efficiency of membrane insertion, in the presence and absence of nanodiscs. A productivity of 0.05 mg, 0.04 mg and 0.1 mg per mL of PURE reaction was obtained for CX_3_CR1, CCR5 and CCR5-Rb, respectively which is sufficient to make a functional and structural analysis (Figure 2).

### 3.2. Membrane Protein Synthesis in Micelle

**Figure 3:**
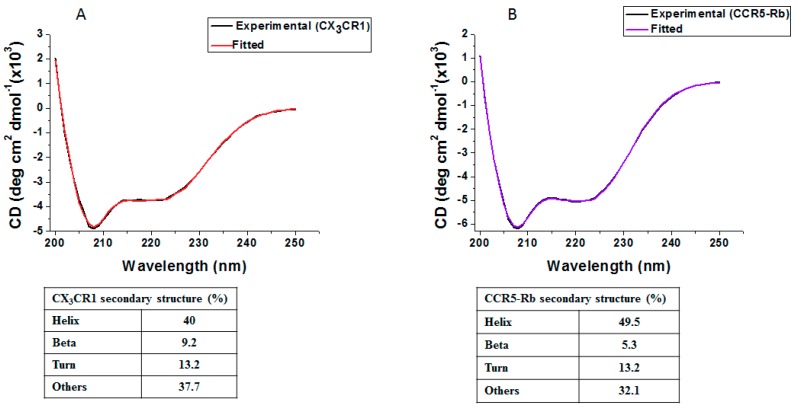
CD measurement of cell-free synthesized GPCRs. Secondary structures of PURE-synthesized CX_3_CR1 (**A**) and CCR5-Rb (**B**) in the presence of a micelle (25 mg/mL brain polar lipid and 75 mg/mL digitonin) were analyzed. For CD measurement, the detergent was exchanged with a buffer containing 20 mM Tris-HCl pH 8.0, 150 mM NaCl, 0.05% DDM and 0.002% CHS during Ni^2+^ column chromatography.

The secondary protein structures of PURE synthesized CX_3_CR1 and CCR5 were estimated by circular dichroism (CD) analysis. As the membrane scaffold protein in nanodisc is alpha helical and interferes with CD measurement of GPCRs, micelles and detergents are usually appropriate for CD analysis. Hence, we synthesized both CX_3_CR1 and CCR5 proteins by the PURE system containing a micelle and the purified proteins were used for CD analysis. The CD spectra showed a minima at around 208 nm and 222 nm indicating a typical characteristic feature of α-helix proteins (Figure 3). The analysis of the α-helix content of CX_3_CR1 and CCR5-Rb proteins in micelles by BeStSel software [34] showed a 40% and 49.5% α-helix content, respectively. The value for CCR5-Rb suggests the formation of a correct secondary structure with the expected 50% α-helix content for GPCRs. In the case of CX_3_CR1, the value is slightly lower and the CD analysis showed slightly higher beta content. We have currently no explanation for such lowered α-helix and increased beta content for CX_3_CR1. Structural information might reveal these aspects in the future.

### 3.3. Membrane Protein Synthesis in Nanodisc and Micelle

**Figure 4:**
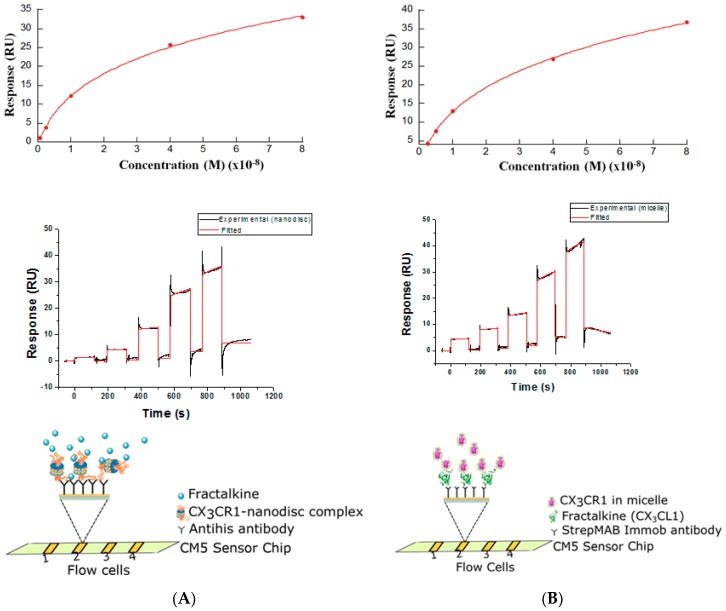
Receptor (CX_3_CR1)-ligand (CX_3_CL1) interaction in different environments. Schematic representation of receptor-ligand interaction in nanodisc (**A**) and micelle (**B**). In both (**A**,**B**), the upper, middle and lower figures correspond to the binding affinity constant, fitted kinetics and schematic representations of the immobilization strategy respectively. In all cases, the analytes at 2.5 nM, 5 nM, 10 nM, 40 nM and 80 nM concentrations were injected to determine the binding affinity constant using single cycle kinetics. For the nanodisc system, CX_3_CL1 protein is denoted as the analyte and for the micelle system, His-tagged CX_3_CR1 is denoted as the analyte.

The structure of CCR5 in complex with the antagonist ligand, maraviroc, has already been solved [25], whereas the structure of CX_3_CR1 has not yet been elucidated. Thus, we determined to further analyze the quality of synthesized CX_3_CR1 by measuring the affinity for its ligand fractalkine (CX_3_CL1) for the future structural analysis of cell-free synthesized GPCRs. For measurement of binding affinity, a nanodisc system and a micelle system were applied. We determined the binding affinity constant of CX_3_CR1-CX_3_CL1 interactions using SPR-based ligand binding assay and evaluated the interaction with the respective ligand by using the affinity values determined by radioligand binding assay as a reference. According to Hoover et al. [35], the fractalkine receptor, CX_3_CR1 binds the chemokine domain of the chemokine ligand with a K_D_ value of 1–4 nM. For the SPR based assay, His-tagged CX_3_CR1 was synthesized by the PURE system in the presence of a nanodisc and the CX_3_CR1-nanodisc complex was immobilized directly on a sensor chip pre-immobilized with the anti-His antibody. A measured binding affinity of 27.8 nM was obtained by injecting a recombinant CX_3_CL1 protein to the sensor chip (Figure 4A). Inversely, to get a larger binding response and for stable and oriented binding, strep-II-tagged ligand CX_3_CL1 was synthesized by the PURE system and immobilized on a sensor chip pre-immobilized with StrepMAB-Immo antibody. A measured binding affinity of 39.4 nM was obtained when purified His-tagged CX_3_CR1 in detergent, which was synthesized by the PURE system containing micelles, was injected into the sensor chip (Figure 4B). This showed that functionally active membrane proteins can be synthesized using nanodiscs or micelles in a PURE system.

### 3.4. Membrane Protein Synthesis in GUV

**Figure 5:**
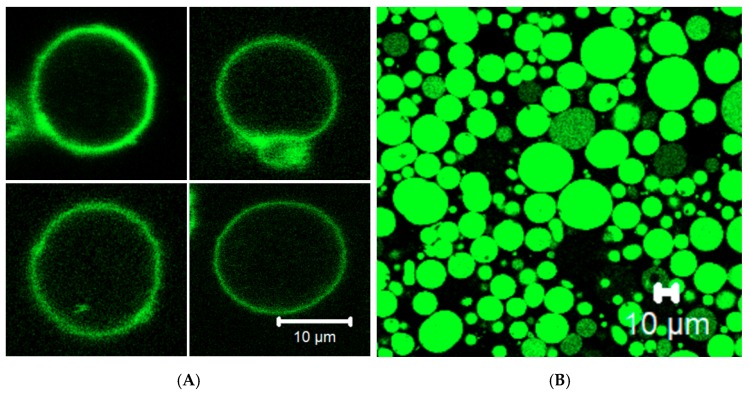
Membrane localization of cell-free synthesized receptor protein. (**A**) Spontaneous membrane integration of CX_3_CR1-sfGFP synthesized inside GUV. Four different representative GUV images taken under a different field of vision were presented. (**B**) sfGFP synthesized inside GUV as a control. Unlike CX_3_CR1-sfGFP, sfGFP was localized in the lumen of the GUVs. The scale bars are 10 µm.

To investigate the membrane localization of cell-free synthesized CX_3_CR1, the protein fused with sfGFP at its C-terminus was synthesized in GUVs containing the PURE system inside and then, its localization was probed by confocal laser microscopy. The result showed that CX_3_CR1 was integrated and localized in the membrane than in the lumen of the GUV (Figure 5A). In contrast, when soluble sfGFP was synthesized in GUV by the PURE system, it was localized in the lumen of the GUV instead of being localized in the membrane (Figure 5B). According to the previous studies [13,15], synthesized CX_3_CR1 might be randomly inserted into GUV membrane, that is, the C-terminus of the protein facing inward and outward as shown in Figure 1C, which should be improved for efficient ligand or antibody binding studies using GUVs. However, these data indicated that appropriate localization of both membrane and soluble proteins can be achieved when they are synthesized in the presence of GUVs. 

### 3.5. Membrane Protein Synthesis in Nanodisc for Structural Analysis

**Figure 6:**
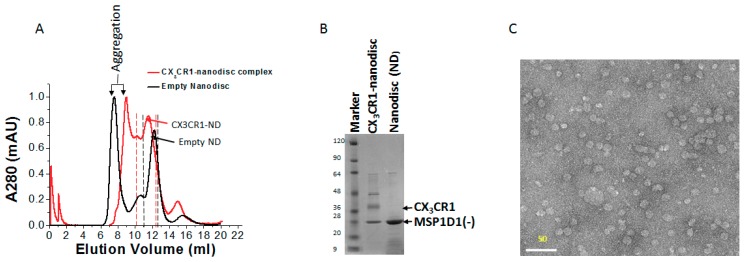
Analysis of CX_3_CR1-nanodisc complex by gel filtration and negative staining. (**A**) Elution pattern of the CX_3_CR1-nanodisc complex (red) and empty nanodisc (black) as observed during the size exclusion chromatography. The peak was normalized to the elution profile of empty nanodisc. (**B**) SDS-PAGE analysis of the elution fraction stained by Coomassie Brilliant Blue (CBB). (**C**) Homogeneity of CX_3_CR1-nanodisc complex analyzed by transmission electron. The scale bar is 50 nm.

Nanodiscs have been used for the structural analysis of membrane proteins by cryo-electron microscopy (cryo-EM) [21,24]. To examine the possibility of cell-free synthesized CX_3_CR1 in nanodiscs, having a defined size that can be controlled by the scaffold protein, for further structural analyses and to confirm the stability and homogeneity of the complex, we carried out gel filtration chromatography and transmission electron microscopic analyses. The purified CX_3_CR1-nanodisc complex with Ni^2+^ affinity purification was further purified with size exclusion chromatography and the purity of the fractions containing the complex was confirmed on a 15% SDS-PAGE. A discrete band of the scaffold protein (MSP1D1-) and CX_3_CR1 at ~25 kDa and ~37 kDa, respectively, were detected (Figure 6A,B). The purified CX_3_CR1-nanodisc complex was further analyzed by transmission electron microscopy and a homogeneous CX_3_CR1-nanodisc complex with a defined size was obtained without an aberrant change in nanodisc size as a result of protein insertion (Figure 6C). In addition, even though it requires further analysis, the SDS-PAGE suggests the insertion of a monomer CX_3_CR1 per nanodisc. Thus, the complex can be used to examine the 3D structure of membrane proteins in their native-like environment.

## 4. Discussion

Cell-free translation systems are used to produce target proteins within a few hours and they are expected to supersede the cell-based expression for functional analysis of membrane proteins due to the advantage in expression and purification processes. In synthetic biology, cell-free systems are indispensable to construct artificial cells capable of mimicking the cellular environment for the investigation of difficult-to-express membrane proteins such as the G-protein coupled receptors [36,37]. In this study, we employed the PURE system to synthesize chemokine G-protein coupled receptors co-translationally in the presence of nanodiscs, micelles and GUVs to enhance their conformational stability and activity.

Producing GPCRs in a cell-free system is inherently challenging due to their aggregation properties and demands in the optimization of the system to obtain active proteins. It was demonstrated by Chi et al. [13] that supplementing the cell-free system with chaperones yields a functional CCR5 with the expected binding affinity. Chemokine receptors and their ligands play a crucial role in the immune system and implicated in various pathophysiological disease [38,39]. However, functional studies of GPCRs in vitro was mainly hampered by precipitation. Thus, translation of CX_3_CR1 and CCR5 chemokine GPCRs in a cell-free system without modification of the system through the addition of lipid bilayers and micelles leads to aggregation and low productivity (Figure 2). The stability and productivity of GPCRs can further be improved by the insertion of a thermostabilizing fragment such as an iron-sulfur redox protein, rubredoxin, at the third intracellular loop of target GPCRs [32]. In our system, insertion of rubredoxin (Rb) to CCR5 improved the productivity in the presence of nanodisc (ND+) by twofold and by fourfold in the absence of nanodisc (ND-) as compared to the yield of CCR5. Whereas the solubility showed no significant improvement despite rubredoxin insertion.

The heptahelical transmembrane of GPCRs is characterized by their α-helix content. Wiktor et al. [40,41] investigated the secondary structure of CCR5 expressed in *E. coli* and confirmed the presence of a minima at around 208 nm and 222 nm which is a typical characteristic of α-helix proteins. As the scaffold protein in nanodiscs contains α-helical structure which interferes with CD measurements, CX_3_CR1 and CCR5 chemokine receptors were expressed by the PURE system supplemented with detergents and micelles. Micelles composed of a brain polar lipid and digitonin detergent were appropriate as most chemokine receptors are expressed in the brain [42,43]. Hence, the brain polar lipid containing a mixture of lipids is expected to provide enhanced stability and activity of CX_3_CR1 and CCR5 chemokine receptors. Therefore, we examined the secondary structure of CX_3_CR1 and CCR5-Rb synthesized by the PURE system in the presence of a micelle and obtained a typical characteristic of folded α-helix identical to the in vivo expressed GPCRs [41]. Though structural analysis of cell-free synthesized CX_3_CR1 is necessary to confirm proper folding, the secondary structure indicates the presence of proper folded CX_3_CR1 and CCR5-Rb synthesized by the PURE system (Figure 3) [40,41]. Nonetheless, the fraction of properly folded receptors in the PURE system is unknown and not yet determined from the secondary structure measurement. Furthermore, NMR analysis of cell-free expressed chemokine GPCRs through 13CH3 methionine will be a substantial approach to better understand the conformational changes of the receptor and will strengthen the secondary structural analysis as it was reported for other classes of GPCRs [36]. 

The interaction of chemokine receptors with their respective ligands determine the functionality of synthesized receptors. Surface plasmon resonance is a label-free assay method used to study ligand-receptor protein interactions and capable of measuring the interactions with high sensitivity and robustness [44]. Shepherd et al. [45] and Rues et al. [22] applied SPR to determine the binding affinity constant of CCR5 in detergent and β1AR reconstituted into nanodiscs. In addition, Shinoda et al. [14] determined the binding affinity constant for cell-free synthesized claudin-4 in a micelle by SPR. Taking advantage of the manipulation of the PURE system reaction condition, we synthesized CX_3_CR1 in the presence of nanodiscs and micelles and measured ligand-receptor interactions.

CX_3_CR1 synthesized by the PURE system was directly immobilized on a sensor chip pre-immobilized with anti-His antibody and a binding affinity comparative to the affinity determined by radioligand binding of in vivo expressed CX_3_CR1 protein was achieved [46]. Nanodiscs enhanced the stability of membrane proteins by lowering the dissociation rate constant and minimizing the disparity in binding affinity constants compared to other methods [47]. Similarly, we succeeded in the synthesis of a chemokine ligand, CX_3_CL1, by the PURE system and immobilized directly on a sensor chip pre-immobilized with StrepMAB-Immo antibody. CX_3_CR1 synthesized by the PURE system containing a micelle, interacted with immobilized ligand and a binding affinity was determined. The binding affinity constants for both systems are outside the range of previously reported values of 1–4 nM by radioligand binding [35]. The possible reason for the discrepancy in binding affinity constant can be explained by the fact that different assay systems or experimental conditions result in a different calculated affinity constant.

Membrane protein analysis can be achieved by using a cell-free system in the presence of nanodiscs, micelles and GUVs. Therefore, our system has a great potential for investigating the functions of membrane proteins including orphan GPCRs. Furthermore, our system can easily be adapted to study oligomerization of GPCRs and other membrane proteins. Most importantly, the synergy of the cell-free system and the nanodisc technology has a tremendous advantage for investigating the structures of membrane proteins in a native-like environment by cryo-electron microscopy (cryo-EM). 

## References

[1] Shen H.H., Lithgow T., Martin L. (2013). Reconstitution of Membrane Proteins into Model Membranes: Seeking Better Ways to Retain Protein Activities. Int. J. Mol. Sci..

[2] Sachse R., Dondapati S.K., Fenz S.F., Schmidt T., Kubick S. (2014). Membrane protein synthesis in cell-free systems: From bio-mimetic systems to bio-membranes. FEBS Lett..

[3] Overington J.P., Al-Lazikani B., Hopkins A.L. (2006). How many drug targets are there?. Nat. Rev. Drug Discov..

[4] Venkatakrishnan A.J., Deupi X., Lebon G., Tate C.G., Schertler G.F., Babu M.M. (2013). Molecular signatures of G-protein-coupled receptors. Nature.

[5] Stevens R.C., Cherezov V., Katritch V., Abagyan R., Kuhn P., Rosen H., Wüthrich K. (2013). The GPCR Network: A large-scale collaboration to determine human GPCR structure and function. Nat. Rev. Drug Discov..

[6] Lappano R., Maggiolini M. (2011). G protein-coupled receptors: Novel targets for drug discovery in cancer. Nat. Rev. Drug Discov..

[7] Noireaux V., Libchaber A. (2004). A vesicle bioreactor as a step toward an artificial cell assembly. Proc. Natl. Acad. Sci. USA.

[8] Fujii S., Matsuura T., Sunami T., Nishikawa T., Kazuta Y., Yomo T. (2014). Liposome display for in vitro selection and evolution of membrane proteins. Nat. Protoc..

[9] Yanagisawa M., Iwamoto M., Kato A., Yoshikawa K., Oiki S. (2011). Oriented reconstitution of a membrane protein in a giant unilamellar vesicle: Experimental verification with the potassium channel KcsA. J. Am. Chem. Soc..

[10] Hamada S., Tabuchi M., Toyota T., Sakurai T., Hosoi T., Nomoto T., Nakatani K., Fujinami M., Kanzaki R. (2014). Giant vesicles functionally expressing membrane receptors for an insect pheromone. Chem. Commun. (Camb.).

[11] Misawa N., Osaki T., Takeuchi S. (2018). Membrane protein-based biosensors. J. R. Soc. Interface.

[12] Shimizu Y., Inoue A., Tomari Y., Suzuki T., Yokogawa T., Nishikawa K., Ueda T. (2001). Cell-free translation reconstituted with purified components. Nat. Biotechnol..

[13] Chi H., Wang X., Li J., Ren H., Huang F. (2015). Folding of newly translated membrane protein CCR5 is assisted by the chaperonin GroEL-GroES. Sci. Rep..

[14] Shinoda T., Shinya N., Ito K., Ishizuka-Katsura Y., Ohsawa N., Terada T., Hirata K., Kawano Y., Yamamoto M., Tomita T. (2016). Cell-free methods to produce structurally intact mammalian membrane proteins. Sci. Rep..

[15] Matsubayashi H., Kuruma Y., Ueda T. (2014). In vitro synthesis of the, *E. coli* Sec translocon from DNA. Angew. Chem. Int. Ed. Engl..

[16] Rues R.B., Dong F., Dötsch V., Bernhard F. (2018). Systematic optimization of cell-free synthesized human endothelin B receptor folding. Methods.

[17] Denisov I.G., Sligar S.G. (2017). Nanodiscs in Membrane Biochemistry and Biophysics. Chem. Rev..

[18] Inagaki S., Ghirlando R., Grisshammer R. (2013). Biophysical characterization of membrane proteins in nanodiscs. Methods.

[19] Yoshiura C., Kofuku Y., Ueda T., Mase Y., Yokogawa M., Osawa M., Terashima Y., Matsushima K., Shimada I. (2010). NMR analyses of the interaction between CCR5 and its ligand using functional reconstitution of CCR5 in lipid bilayers. J. Am. Chem. Soc..

[20] Gao Y., Cao E., Julius D., Cheng Y. (2016). TRPV1 structures in nanodiscs reveal mechanisms of ligand and lipid action. Nature.

[21] Mi W., Li Y., Yoon S.H., Ernst R.K., Walz T., Liao M. (2017). Structural basis of MsbA-mediated lipopolysaccharide transport. Nature.

[22] Rues R.B., Dötsch V., Bernhard F. (2016). Co-translational formation and pharmacological characterization of beta1-adrenergic receptor/nanodisc complexes with different lipid environments. Biochim. Biophys. Acta.

[23] Klammt C., Srivastava A., Eifler N., Junge F., Beyermann M., Schwarz D., Michel H., Doetsch V., Bernhard F. (2007). Functional analysis of cell-free-produced human endothelin B receptor reveals transmembrane segment 1 as an essential area for ET-1 binding and homodimer formation. FEBS J..

[24] Efremov R.G., Gatsogiannis C., Raunser S. (2017). Lipid Nanodiscs as a Tool for High-Resolution Structure Determination of Membrane Proteins by Single-Particle Cryo-EM. Methods Enzymol..

[25] Tan Q., Zhu Y., Li J., Chen Z., Han G.W., Kufareva I., Li T., Ma L., Fenalti G., Li J. (2013). Structure of the CCR5 chemokine receptor-HIV entry inhibitor maraviroc complex. Science.

[26] Shimizu Y., Kuruma Y., Kanamori T., Ueda T. (2014). The PURE system for protein production. Methods Mol. Biol..

[27] Ritchie T.K., Grinkova Y.V., Bayburt T.H., Denisov I.G., Zolnerciks J.K., Atkins W.M., Sligar S.G. (2009). Reconstitution of membrane proteins in phospholipid bilayer nanodiscs. Methods Enzymol..

[28] Bayburt T.H., Sligar S.G. (2010). Membrane protein assembly into Nanodiscs. FEBS Lett..

[29] Denisov I.G., Grinkova Y.V., Lazarides A.A., Sligar S.G. (2004). Directed self-assembly of monodisperse phospholipid bilayer Nanodiscs with controlled size. J. Am. Chem. Soc..

[30] Lamichhane R., Liu J.J., Pauszek R.F., Millar D.P. (2017). Fluorophore Labeling, Nanodisc Reconstitution and Single-molecule Observation of a G Protein-coupled Receptor. Bio-Protocol.

[31] Shimizu Y., Ueda T. (2010). PURE technology. Methods Mol. Biol..

[32] Chun E., Thompson A.A., Liu W., Roth C.B., Griffith M.T., Katritch V., Kunken J., Xu F., Cherezov V., Hanson M.A. (2012). Fusion partner toolchest for the stabilization and crystallization of G protein-coupled receptors. Structure.

[33] Henrich E., Dötsch V., Bernhard F. (2015). Screening for lipid requirements of membrane proteins by combining cell-free expression with nanodiscs. Methods Enzymol..

[34] Micsonai A., Wien F., Kernya L., Lee Y.H., Goto Y., Réfrégiers M., Kardos J. (2015). Accurate secondary structure prediction and fold recognition for circular dichroism spectroscopy. Proc. Natl. Acad. Sci. USA.

[35] Hoover D.M., Mizoue L.S., Handel T.M., Lubkowski J. (2000). The Crystal Structure of the Chemokine Domain of Fractalkine Shows a Novel Quaternary Arrangement. J. Biol. Chem..

[36] Shilling P.J., Bumbak F., Scott D.J., Bathgate R.A.D., Gooley P.R. (2017). Characterisation of a cell-free synthesised G-protein coupled receptor. Sci. Rep..

[37] Cook B.L., Steuerwald D., Kaiser L., Graveland-Bikker J., Vanberghem M., Berke A.P., Herlihy K., Pick H., Vogel H., Zhang S. (2009). Large-scale production and study of a synthetic G protein-coupled receptor: Human olfactory receptor 17-4. Proc. Natl. Acad. Sci. USA.

[38] Ren H., Yu D., Ge B., Cook B., Xu Z., Zhang S. (2009). High-level production, solubilization and purification of synthetic human GPCR chemokine receptors CCR5, CCR3, CXCR4 and CX3CR1. PLoS ONE.

[39] Miller M.C., Mayo K.H. (2017). Chemokines from a Structural Perspective. Int. J. Mol. Sci..

[40] Wiktor M., Morin S., Sass H.J., Kebbel F., Grzesiek S. (2013). Biophysical and structural investigation of bacterially expressed and engineered CCR5, a G protein-coupled receptor. J. Biomol. NMR.

[41] Corin K., Baaske P., Ravel D.B., Song J., Brown E., Wang X., Geissler S., Wienken C.J., Jerabek-Willemsen M., Duhr S. (2011). A robust and rapid method of producing soluble, stable, and functional G-protein coupled receptors. PLoS ONE.

[42] Sorce S., Myburgh R., Krause K.H. (2011). The chemokine receptor CCR5 in the central nervous system. Prog. Neurobiol..

[43] Hulshof S., van Haastert E.S., Kuipers H.F., van den Elsen P.J., De Groot C.J., van der Valk P., Ravid R., Biber K. (2003). CX3CL1 and CX3CR1 expression in human brain tissue: Noninflammatory control versus multiple sclerosis. J. Neuropathol. Exp. Neurol..

[44] Chu R., Reczek D., Brondyk W. (2014). Capture-stabilize approach for membrane protein SPR assays. Sci. Rep..

[45] Shepherd C.A., Hopkins A.L., Navratilova I. (2014). Fragment screening by SPR and advanced application to GPCRs. Prog. Biophys. Mol. Biol..

[46] Harrison J.K., Fong A.M., Swain P.A., Chen S., Yu Y.R., Salafranca M.N., Greenleaf W.B., Imai T., Patel D.D. (2001). Mutational analysis of the fractalkine chemokine domain. Basic amino acid residues differentially contribute to CX3CR1 binding, signaling, and cell adhesion. J. Biol. Chem..

[47] Guo D., Heitman L.H., IJzerman A.P. (2017). Kinetic Aspects of the Interaction between Ligand and G Protein-Coupled Receptor: The Case of the Adenosine Receptors. Chem. Rev..

